# Massive Fetomaternal Hemorrhage Caused by an Intraplacental Choriocarcinoma: A Case Report

**DOI:** 10.1155/2010/767218

**Published:** 2010-03-03

**Authors:** Anna-Karina Aaris Henningsen, Lisa Leth Maroun, Hanne Havsteen, Jens Svare

**Affiliations:** ^1^Department of Obstetrics and Gynecology, Glostrup Hospital, University of Copenhagen, Nodre Ringvej 37, 2600 Glostrup, Denmark; ^2^Fertility Clinic, Rigshospitalet, University of Copenhagen, Blegdamsvej 9, 2100 Copenhagen, Denmark; ^3^Department of Pathology, Rigshospitalet, University of Copenhagen, Blegdamsvej 9, 2100 Copenhagen, Denmark; ^4^Department of Oncology, Herlev Hospital, University of Copenhagen, Herlev Rinvej 75, 2730 Herlev, Denmark

## Abstract

*Background*. Intraplacental choriocarcinoma is a rare but highly malignant trophoblastic neoplasm. When found near term the risk of maternal metastasis is high because of the late diagnosis. *Case*. We describe a case of an intraplacental choriocarcinoma diagnosed postpartum after a near-term delivery of a severely anemic infant. A fetomaternal hemorrhage resulted in a hemoglobin concentration in the infant of only 2,1 g/dL. Neither mother nor child showed signs of metastatic disease. The macroscopic examination showed a hydropic placenta weighing more than 1 kilogram. Microscopy showed an intraplacental choriocarcinoma 3 cm in diameter. The tumor had infiltrated the maternal basal plate. *Conclusion*. Fetomaternal bleeding is a rare form of presentation of choriocarcinoma but its presence should always warrant detailed examination of placenta, mother, and infant.

## 1. Introduction

Gestational trophoblastic disease (GTD) covers a spectrum of diseases from benign trophoblastic lesions over the premalignant abnormal conceptions, the hydatidiform moles, either partial or complete, to the more rare malignant forms, the invasive mole, the placental site and epithelioid trophoblastic tumors and choriocarcinoma [1]. 

Choriocarcinoma is a rare but highly malignant tumor, the most aggressive form of gestational trophoblastic disease [2] and intraplacental choriocarcinoma is a rare variant accounting for no more than approximately 0.04% of gestational trophoblastic disease [3]. The risk of gestational trophoblastic disease is increased in older and multiparous women and in very young women [4]. 

Choriocarcinoma following a live birth is reported to have an incidence of 1 in 50 000 births and to be associated with a poor prognosis, as a delay in diagnosis increases the risk of widespread dissemination, both maternal and fetal [5–7]. Massive feto-maternal hemorrhage can cause fetal distress and death at term and the possibility of intraplacental choriocarcinoma should always be considered in these cases [5].

We describe a case of an intraplacental choriocarcinoma causing massive feto-maternal bleeding in a near term pregnancy with signs of fetal distress.

## 2. Case Report

The mother was a 47-year-old woman, para 7, originally from Somalia. The father was 71 years old. The mother presented at 35 + 6 weeks of gestational age after 3 days of decreased fetal movements. Cardiotocography (CTG) was severely pathologic, showing a preterminal pattern. Emergency caesarean section was performed. 

The antenatal course had been uneventful up until the last few weeks before delivery when the mother had started to complain about fatigue and palpitations. Four days before delivery the mother had fainted and was examined at the maternity ward. Her level of hemoglobin and the electrocardiography (ECG) were normal, as was the CTG.

The newborn weighed 2800 gram and had Apgar scores of 0 at 1 minute, 4 at 2 minutes and 7 at 5 minutes. At birth a sample of umbilical arterial blood showed metabolic acidosis. Blood glucose was normal.

At first there were vital signs but soon the child turned pale, weak and was without cardiac activity. Cardiopulmonary resuscitation was initiated. The infant was found to be severely anemic, the hemoglobin concentration in the umbilical artery was 2.1 g/dL. She was immediately treated with blood transfusion.

Hereafter she was admitted to the neonatal intensive care unit, where she was intubated, ventilated and treated with nitrous oxide (NO) because of pulmonary hypertension. 

Echocardiography showed severe distention of the right side of the heart as well as a small apical ventricular septum defect (VSD). Both ultrasound of the cerebrum and electro-encephalogram (EEG) were normal. Because of neonatal sepsis the infant was treated with a broad spectrum of intravenous antibiotics.

The infant remained hospitalized for 1 month. The serum levels of both alfa-foetoprotein and hCG were at all times normal why no ultrasound, X-ray or CT scans were performed in search for metastases. The infant showed no signs of neurological damage. After discharge she was seen regularly in the out-patient clinic with measurements of serum levels of alfa-foetoprotein and hCG as well of monitoring of her neurological development. The mother was simultaneously examined because of the risk of widespread disease. Chest X-ray revealed no metastatic lesion, neither did ultrasound or CT scan of the abdomen.

The mother was referred to a regional oncology center where serial serum concentrations of hCG and alfa-foetoprotein were measured, for the first month twice a week and hereafter twice a month until 2 months after normalization of the serum hCG level. From there on the mother's serum hCG will be measured every second month until the level of serum hCG has been normal for 14 months. On the 17th day postpartum the hCG was 510 iU/L dropping to 16 iU/L 36 days postpartum. Since there were no signs of dissemination, the mother was not treated with chemotherapy. Six months postpartum, both mother and child are doing well, showing no signs of malignant disease.

## 3. Pathological Findings

**Figure 1:**
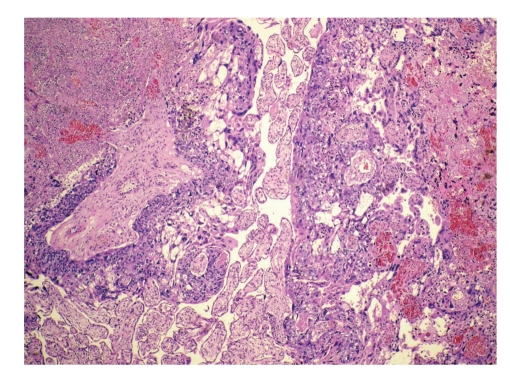
Intraplacental choriocarcinoma growing out from stem villi sharply demarcated from surrounding normal villi (H&E ×40).

**Figure 2:**
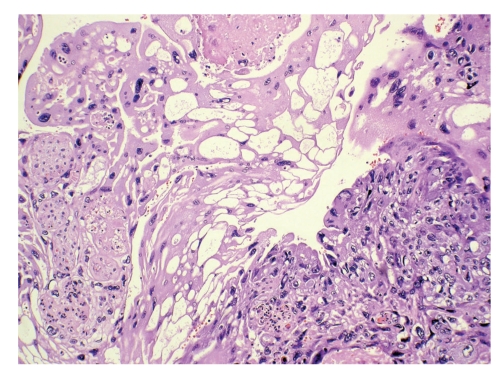
Biphasic tumor tissue with neoplastic syncytiotrophoblast to the left and neoplastic cytotrophoblast to the right (H&E ×200).

The placenta was large, pale and edematous. It weighed 1060 g. On initial gross examination performed by a pathologist without knowledge of the clinical circumstances some intraplacental hematomas were found. Histologically the placenta was characterized by edematous immature villi and a number of intraplacental hematomas (intervillous thrombi). The fetal blood showed a significant increase in nucleated fetal red blood cells as a response to fetal anemia. Before the final report was issued the presence of severe feto-maternal hemorrhage was communicated to the pathologist. The placenta was reexamined and additional sections submitted. On gross reexamination, an indistinct 3 cm poorly demarcated soft hemorrhagic area was noted. Histologically this area showed a biphasic tumor tissue growing out from stem villi (Figure 1) and consisting of alternating areas of highly atypical and pleomorphic syncytiotrophoblast and cytotrophoblast (Figure 2). The tumor tissue showed an irregular but sharply defined border towards the surrounding nonneoplastic placental tissue (Figure 1). Small tumor satellites were present near the main tumor mass and the tumor had infiltrated the maternal basal plate and was present in the deepest aspect of the specimen. The tumor was associated with significant hemorrhage.

In this case, if the clinical information had not been communicated to the pathologist, the intraplacental choriocarcinoma would not have been found.

## 4. Discussion

Intraplacental choriocarcinoma is a very rare and aggressive disease, seldom diagnosed at the time of delivery [7]. Earlier studies have found that 4% of patients with persistent gestational trophoblastic disease are diagnosed with choriocarcinoma following term pregnancy [7]. The majority of patients with choriocarcinoma present weeks or months postpartum with either vaginal bleeding, caused by vaginal or uterine metastases or symptoms related to metastases in lungs, brain, breast or liver [6]. 60% to 87% of patients with postterm choriocarcinoma have been found to have metastases at the time of diagnosis and the overall survival is around 86% in patients with a postpartum choriocarcinoma [3, 7].

The prevalence of choriocarcinoma is low, but it is most often seen in women less than 15 years or more than 45 years of age, as well as in multiparous women [4, 5, 8]. Both advanced maternal age and multiparity was present in our case of gestational choriocarcinoma. 

Choriocarcinomas can be preceded by a complete mole, a miscarriage or a normal pregnancy. Cases have been described where choriocarcinoma is preceded by a partial mole although this is more uncommon [2]. When preceeded by a normal pregnancy the outcome is often intrauterine death. Very often, intraplacental choriocarcinoma is associated with maternal disseminated disease and more rarely dissemination in the fetus as well [7]. When choriocarcinoma is discovered with term pregnancy the risk of widespread metastases is high. Although the condition is extremely uncommon, it is potentially treatable. In the case of early diagnosis and appropriate chemotherapy, prognosis is good [7].

Indications for detailed examination or sometimes re-examination of the placenta are evidence of maternal metastatic disease or complications such as intrauterine fetal death, stillbirth or as in our case fetomaternal hemorrhage [9].

The causes of fetomaternal hemorrhage are numerous, including traumas to the uterus, placental chorangiomas and procedures such as amniocentesis or external cephalic version of the baby. In 1962 Benson et al. [10] were the first to describe a case of massive fetomaternal hemorrhage as a complication of choriocarcinoma. Feto-maternal hemorrhage is a very rare form of presentation of choriocarcinoma but whenever seen the question of malignancy should be raised.

In our case the diagnosis of intraplacental choriocarcinoma was not found during the initial pathological examination of the placenta, but the critical state of the newborn because of significant feto-maternal hemorrhage led to the re-examination of the placenta and to the discovery of the malignancy.

Our case demonstrates that feto-maternal hemorrhage should always be followed by detailed examination of the placenta as well as serial serum-hCG monitoring of both mother and child, in an attempt to diagnose early signs of possible malignant dissemination.

The macroscopic diagnosis of placental tumor is often difficult because of its innocuous gross appearance, small size and the resemblance to benign lesions, such as infarction and intraplacental hematoma [3, 9]. This case clearly illustrates the importance of clinico-pathological correlation and we presume that the prevalence of intraplacental choriocarcinoma is most probably notably higher than previously reported.
